# The Downstream Effects of Teacher Well-Being Programs: Improvements in Teachers' Stress, Cognition and Well-Being Benefit Their Students

**DOI:** 10.3389/fpsyg.2021.689628

**Published:** 2021-07-01

**Authors:** Annemaree Carroll, Ashley York, Sam Fynes-Clinton, Emma Sanders-O'Connor, Libby Flynn, Julie M. Bower, Kylee Forrest, Maryam Ziaei

**Affiliations:** ^1^School of Education, The University of Queensland, Brisbane, QLD, Australia; ^2^School of Psychology, The University of Queensland, Brisbane, QLD, Australia; ^3^Centre for Advanced Imaging, The University of Queensland, Brisbane, QLD, Australia

**Keywords:** teacher well-being, student well-being, teacher stress, teacher burnout, academic self-perceptions, classroom environment

## Abstract

Quality interventions addressing the important issue of teacher stress and burnout have shown promising outcomes for participating teachers in terms of decreased distress, improved well-being and increased commitment to their jobs. Less is known however about whether such interventions also benefit students. The present study investigated the downstream effects for a completer sample of 226 primary and high school students after their teachers (*n* = 17) completed one of two 8-week stress reduction interventions. The relationships between change in teacher self-reported distress and burnout after completing the interventions, and change in students' self-reported well-being, academic self-perceptions, and perceptions of classroom environment were explored. A secondary aim of this study was to assess whether changes in teachers' cognitive flexibility mediated the relationship between teacher and student self-report outcomes. Results of correlational and multi-level mediation analyses showed that changes to teachers' self-reported distress and burnout affected multiple facets of students' well-being and the academic environment. Specifically, reductions in teachers' self-reported distress and burnout were related to students' improved perceptions of their teachers' support in the classroom. Reductions in teachers' personal and work-related burnout correlated with greater increases of academic self-perception in students. Contrary to predictions, cognitive flexibility in teachers did not mediate the relationship between these student and teacher measures. These findings indicate important downstream benefits for students and highlight the broader value of stress-reduction and well-being programs for teachers.

## Introduction

The teaching profession is associated with high stress (Skaalvik and Skaalvik, 2010; Fisher, 2011; Aloe et al., 2014; Chirico et al., 2020) and work burnout (Lawrence et al., 2019) which may lead to reduced job satisfaction (Wang et al., 2015) and a greater number of teachers leaving the workforce (Goddard et al., 2006; Chirico, 2017a). The Covid-19 pandemic has underscored the vital importance of teachers to society, with teachers worldwide stepping up to an incredible challenge as essential frontline workers continuing to support and educate children in highly uncertain times. The pandemic has highlighted the critical role that teachers play in meeting both the academic and non-academic needs of students with prolonged school closures adding to the burden on teachers to rapidly upskill their digital literacy and support the learning and well-being of students remotely (Hoffman and Miller, 2020; Lucas et al., 2020). This disruptive era in schooling has added to the stress and burden on teachers. It has been estimated that ~40% of Australian teachers leave the profession within their first five years (Weldon, 2018). With attrition rates soaring (Arnup and Bowles, 2016), numerous studies have sought to identify the causes of teacher stress and aimed to identify ways of addressing this issue (e.g., Collie et al., 2012; Carroll et al., 2020). However, few studies have explored the downstream effects of high stress in teachers on student well-being; even fewer have explored the potential benefits for students when their teachers engage in stress-reduction programs, which was the primary aim of the present study.

Teacher stress has been defined as “the experience by a teacher of unpleasant, negative emotions, such as anger, anxiety, tension, frustration or depression, resulting from some aspect of their work” (Kyriacou, 2001, p. 28). Burnout has been conceptualized as “a state of physical, emotional, and mental exhaustion that results from long-term involvement in work situations that are emotionally demanding” (Schaufeli and Greenglass, 2001, p. 501). Chronic stress is implicit in the development of burnout (e.g., Brackett et al., 2010; Vesely et al., 2013), is associated with short- and long-term mental and physical health problems (Steinhardt et al., 2011) and plays a large role in attrition from the profession (Struyven and Vanthournout, 2014; Chirico et al., 2020). A broad range of factors from multiple domains can contribute to the development of teacher stress, including: systemic (i.e., government and department policy), cultural (i.e., leadership style, school culture, level of autonomy, administrative and collegial support, workload), relational (e.g., students, parents, classroom environment) and intrapersonal (e.g., emotion regulation, coping style, personality) factors (e.g., Carroll et al., 2020). Stress is known to decrease well-being in teachers (Griffith et al., 1999; Collie et al., 2012; Chirico, 2017a) and has been suggested to affect student well-being and academic performance (Durlak et al., 2011; Ramberg et al., 2020).

In the present study, well-being is defined as an intricate combination of emotional, social and mental health factors which contribute to one's sense of purpose in life and affective state (Deci and Ryan, 2008). Teacher well-being is known to positively influence student outcomes (Baumeister et al., 2003) and school climate (Ross et al., 2012). Although numerous studies have explored the effectiveness of targeted social, behavioral, and emotional intervention programs (e.g., Durlak et al., 2011; Hwang et al., 2017; Goldberg et al., 2019), these studies have only explored outcomes in teachers or students independently. There is a lack of research exploring the flow-on or downstream effects of student-focused interventions on teachers' well-being, and teacher-focused interventions on student well-being.

Studies that have attempted to identify precursory factors contributing to student well-being and academic performance have shown that there is a dynamic relationship between teachers, students, and classroom environment outcomes (Jennings and Greenberg, 2009; Durlak et al., 2011; Collie et al., 2012; Oberle and Schonert-Reichl, 2016). When teachers are functioning well, there are a wealth of benefits for teachers themselves and their students. For example, one study showed that teachers who scored well across multiple domains of well-being were more committed to their school, and more satisfied with their health, life and chosen occupation (Kern et al., 2014). Other studies have demonstrated the link between positive teacher well-being and higher academic performance in students (e.g., Caprara et al., 2006; Duckworth et al., 2009), improved teaching practices and student learning (e.g., Turner and Thielking, 2019), and improved student-teacher relationships (Carroll et al., under review).

Conversely, Jennings and Greenberg (2009) highlight the cyclical relationship between teachers' stress levels, teachers' competence in behavior management, and students' experiences of the classroom. Oberle and Schonert-Reichl (2016) directly linked teachers' occupational stress levels to students' physiological stress regulation, finding that higher levels of teacher burnout significantly predicted high production of the stress hormone, cortisol, in students. The authors discuss the connection between teacher burnout and student stress hormone release, within the framework of stress-contagion theory (Wethington, 2000). Stress-contagion theory posits that within a social context (i.e., the classroom) stress from one individual (i.e., the teacher) can “cross-over” and influence other individuals (i.e., the student). Together, these studies suggest that the relationship between teacher and student well-being is complex and bidirectional.

One possibility for the relationship between teacher and student well-being is that cognitive performance in teachers is a catalyst for change in the classroom environment. Evidence indicates that stress and burnout can have a negative influence on cognitive performance across various domains including cognitive flexibility and cognitive control (Averill, 1973; Steinhauser et al., 2007; Gabrys et al., 2018). Chronic stress is also associated with an elevated incidence of unpleasant emotions and affective disorders, particularly anxiety, depression and somatization (e.g., Monat and Lazarus, 1991; Bourbonnais et al., 1999; Godin et al., 2005). Research has consistently shown that elevated emotional states impair an individual's ability to engage in executive functioning tasks such as switching attention, inhibition, and goal orientated behavior (Baumeister et al., 2003; Schwabe et al., 2012). Given that stress impacts cognitive control and flexibility, and that teachers require strong skills in these domains, students may be influenced by teacher stress at least partially because there is a change in teacher's cognitive performance which influences the classroom environment. It is possible that enhanced cognitive performance in teachers, associated with improved well-being, is a mechanism through which sustainable change in student well-being and the classroom environment occurs.

The data used in the present study were collected as part of a larger intervention study exploring the effect of stress-reduction programs for teachers (Carroll et al., under review). The programs explored in the intervention study were the Mindfulness Based Stress Reduction Program (Kabat-Zinn, 1990) and the Health Enhancement Program (MacCoon et al., 2012), both of which were adapted so that examples, discussion points, and strategies were specifically relevant to the working lives of teachers. Both of these interventions resulted in equivalent significant and sustained improvements in teachers' work stress, burnout, psychological distress, emotion regulation, mindfulness, well-being, cognitive flexibility and working memory after completing the programs. In other words, these programs were highly effective for reducing stress and burnout, and for improving cognition and well-being for teachers. In the present study, we were interested in whether change in teachers' stress, cognition and well-being had downstream effects on their students, particularly on students social and emotional well-being, academic self-perceptions, and classroom environment. The following predictions were made:

Prediction 1: Reductions in teacher distress would positively influence student well-being, academic self-perceptions, and their evaluation of the classroom environment.

Prediction 2: Reductions in teacher burnout would positively influence student well-being, academic self-perceptions, and their evaluation of the classroom environment.

A secondary aim of the present study was to explore whether cognitive flexibility might mediate the relationship between teacher distress and burnout with student well-being, academic self-perceptions, and evaluation of classroom environment.

## Materials and Methods

### Participants

A total of 19 teachers (16 females and 3 males) and their students (*n* = 278; 157 females and 122 males) participated in the present study. Thirty students were excluded (from two teacher samples) because they withdrew before the second data collection time point, leaving a final completer sample of 17 teachers and 226 students. Demographics of the teacher and student samples are presented in Table 1. Teacher participants were recruited via an advertisement emailed to school principals in Brisbane, Australia, who then forwarded the invitation to their school staff. Teachers who expressed interest in the study via email were followed up with a screening phone call and online form to assess eligibility. Selection criteria for the teacher sample included: being a current practicing teacher; experiencing work-related stress; having no current neuropsychological disorders; not using psychotropic medication or illicit substances; not currently engaging in regular vigorous exercise or regular meditation practice; and being willing and able to commit to the time and homework requirements of the study. Student participants were recruited via their participating classroom teacher.

**Table 1:** Student Demographics (as reported at study commencement).

**Sample**	**Demographic**	**Mean (Standard Deviation)**	**Range**
Teachers	Age	48.9 years (13.0)	25–69 years
	Years teaching	21.3 years (12.9)	1.5–46 years
Students	Age	11.4 years (2.21)	8–17 years
	Grade	4.12 (3.09)	Grades 1–9

### Teacher Measures

#### Teacher Self-Report Outcome Measures

*The Depression Anxiety Stress Scale - 21* (DASS-21; Lovibond and Lovibond, 1995) was used as a measure of psychological distress. It comprises three, 7-item subscales: Depression, Anxiety, and Stress. Respondents indicate how often they have experienced emotional and physical symptoms of distress during the past week according to a four-point scale ranging from 0 (did not apply to me at all) to 3 (applied to me very much or most of the time). The DASS-21 has been validated in numerous non-clinical populations and has been shown to have good psychometric properties (Dreyer et al., 2019). Due to the high comorbidity of anxiety, depression, and stress, a total DASS score was calculated as a composite measure of negative emotional symptoms (Watson and Clark, 1984; Lovibond and Lovibond, 1995), referred to herein as Distress. The total DASS score ranges from 0 to 126, with higher scores indicating a greater degree of Distress.

The *Copenhagen Burnout Inventory (CBI;* Kristensen et al., 2005) is a 19-item scale assessing symptoms of burnout for individuals working in the human service sector. The CBI conceptualizes burnout as the degree of physical and psychological fatigue and exhaustion experienced by a person and assesses three domains: Personal Burnout (general; 6 items), Work Burnout (specifically attributed to work; 7 items), and Student-Related Burnout (specifically attributed to work with students; 6 items). Responses are indicated on a five-point scale comprised of: never/almost never or to a very low degree (0%); seldom or to a low degree (25%); sometimes or somewhat (50%); often or to a high degree (75%); or always or to a very high degree (100%). Scores for each subscale are calculated as averages, with a possible range of 0–100.

#### Teacher Cognitive Control Measures

Participants completed a subset of tests from the Cambridge Neuropsychological Test Automated Battery (CANTAB, 1999). For the purpose of this study, the Attention Switching Task was examined as a possible mediator. The Attention Switching Task measures executive functioning and the ability to manage conflicting information through inhibitory control and cognitive flexibility. This variable will be herein referred to as Cognitive Flexibility. Tasks were presented on an iPad with instructions embedded into the software to avoid experimenter error. On repeated administration of the Attention Switching Task, a varied but equivalent version of the tasks was used to eliminate practice effects. Participants were first trained to respond to an arrow on the screen by pressing the button (left or right) that corresponded with the direction of the arrow. During the next training phase, the arrow appeared on either the right or left side of the screen and a cue for type of response was given at the top of the screen (i.e., either “SIDE” or “DIRECTION”). During SIDE cued trials, participants were required to press the button that corresponded with the side of the screen that the arrow appeared on; in the DIRECTION cued trials, participants were asked to press the button that corresponded with the direction of the arrow regardless of the side of screen on which it was presented. In total, 160 trials with an equal number of matching or mismatching direction and location cues were presented in a randomized order. The arrow and response cue were presented for 500 ms followed by a 2000 ms response screen. Teachers' reaction times (RT) and accuracy were recorded as the dependent variables for Cognitive Flexibility, with lower RTs and higher accuracy indicating superior performance. Full public access to the task descriptions is available at (http://www.cambridgecognition.com/cantab/cognitive-tests).

### Student Measures

Students completed several well-established, psychometrically sound measures designed to assess emotional well-being, psychological functioning, perception of their classroom environment, and academic achievement. Student measures were completed at two time points: before and after teachers completed the stress-reduction programs, approximately 10 weeks apart.

#### Student Self-Report Outcome Measures

The *Strengths and Difficulties Questionnaire* (SDQ; Goodman, 1997) is a 25-item scale yielding subscale scores for students' emotional symptoms, conduct problems, hyperactivity, peer problems and prosocial behaviors, as well as a total difficulties score. Of interest for the present study was the Total Difficulties score which is generated by summing scores from all the scales except the prosocial scale, with a range of 0 to 40. Responses are made on a 3-point scale (1 = not true; 2 = somewhat true; 3 = certainly true), with higher scores indicating more emotional and behavioral difficulties on the SDQ.

The *Warwick-Edinburgh Mental Well-being Scale* (WEMWBS; Tennant et al., 2007) is a 14-item scale of mental well-being covering subjective well-being and psychological functioning. All items are worded positively and address aspects of positive mental health. Participants respond to the items using a five-point Likert scale ranging from (1 = none of the time to 5 = all of the time). Responses are based on the participant's feelings over the previous two weeks. Higher levels of positive mental well-being are indicated by higher scores (Tennant et al., 2007).

*Classroom Environment Scale* (CES; Trickett and Moos, 1973; Moos and Trickett, 1974; Fraser and Fisher, 1983) is designed to measure students' perceptions of the classroom environment and the relationship between student affect and school outcomes. In the present study we used a 24-item variant of the CES which included the following subscales: Involvement, Affiliation, Teacher Support, Task Orientation, Order, Organization and Rule Clarity. Responses were made by indicating “yes” or “no” as to whether each statement was true for the participant. Higher scores indicate more “yes” responses agreeing with positively-phrased items including the examples; “*The teacher goes out of his/her way to help students”* and “*Most students in this class really pay attention to what the teacher is saying”;* and fewer “no” responses to negatively-phrased items such as “*Rules in this class seem to change a lot.”*

*Academic Self-Perception* (ASP) subscale (McCoach and Siegle, 2003a) of the School Attitude Assessment Survey-Revised (SAAS-R; McCoach and Siegle, 2003b) measures students' positive self-perception regarding their own academic ability. The ASP subscale has 5-items and participants are required to make responses on a 7-point Likert scale (1 = *strongly disagree*; 7 = *strongly agree*), with items such as; “*I am confident in my ability to succeed in school”*. Scores are recorded as total scores with a possible range of 1–35, with higher scores indicating a more positive self-perception about academic ability.

### Procedure

Approval for this study was obtained from the Human Research Ethics Committee of the administering organization. Gatekeeper approval to contact schools was also provided from the Department of Education, Catholic Education, and Independent Public Schools in the large metropolitan city in which the study was conducted. Written, informed consent to take part in the study was obtained from the participating school principals, all teacher and student participants, and students' parents/guardians.

All participating teachers took part in one of two eight-week teacher-stress reduction programs: The Mindfulness Based Stress Reduction (MBSR) Program, developed by (Kabat-Zinn, 2003) or the Health Enhancement Program (HEP), originally developed by MacCoon et al. (2012). Both programs were adapted to be particularly relevant for teachers (Carroll et al., under review). A matched samples approach was used to allocate participants to the interventions. As reported in Carroll et al. (under review), both interventions led to a range of significant and sustained improvements for teachers that were maintained at 5-month follow-up. This included significant reductions in teachers' levels of distress and burnout (as measured by the DASS and CBI), and improvements in cognitive flexibility (as measured by the Attention Switching Task of the CANTAB). No differences between the MBSR and HEP interventions were found on any measure of interest for the present study. As such, for the purposes of investigating the downstream influence of teacher stress and burnout on students in the present study, intervention type was not investigated separately. Rather, teacher data were collapsed across both intervention groups.

Student and teacher data used in the present study were collected at two time points: within 4 weeks of the teachers beginning the intervention programs (pre-intervention) and within 4 weeks after the teachers had completed the intervention programs (post-intervention). Students completed their questionnaires during a convenient period of class time with the assistance of research staff. As described in Carroll et al. (under review), teachers completed all their assessment at the administering institution.

### Data Analysis

All data were converted to difference scores reflecting longitudinal change in outcomes from pre- to post-intervention. Bivariate correlations were calculated between student outcome measures and teacher self-reported Distress (Total DASS score) and Burnout (Step 1). Correlations were then calculated between related student and teacher measures from Step 1 and Cognitive Flexibility in teachers (Step 2). Fully inter-correlated teacher and student measures (identified through Steps 1 and 2) were included in a multi-level mediation analysis (Step 3) to explore whether changes in teacher Cognitive Flexibility mediate the relationship between teacher Distress and Burnout and student difficulties, well-being, academic self-perceptions, and classroom environment.

Multilevel modeling is the preferred method for examining mediation effects when observations are nested (Rockwood, 2017). In the present study, data were collected within a two-level hierarchical structure with lower (student) level variables as well as upper (teacher) level variables. Thus, the student data are not independent of the teacher data and if treated independently, could lead to an underestimation of standard errors in the model (Snijders and Bosker, 1999). Multilevel mediation accounts for the dependence of data within groups and allows for the investigation of top-down influences of upper level (teacher) variables on lower level (student) outcomes (Kenny et al., 1998).

In the present study, an upper multi-level mediation model was tested with teacher self-report measures included as predictors, student self-report measures included as outcomes, and measures of teacher Cognitive Flexibility tested as mediators (i.e., 2-2-1 mediation model; similar labels have been used by Krull and MacKinnon, 2001; Bauer et al., 2006). To assess the indirect pathways (i.e., mediation), multi-level mediation analyses were conducted in R and linear mixed effects models were calculated using the lmer function from the lme4 package (Bates et al., 2015). Statistical significance of the regression coefficients was calculated using the lmerTest package (Kuznetsova et al., 2017). Due to the small number of teachers (*n* = 17), the models were fit with ReML (restricted maximum likelihood estimation; Chu et al., 2011; Bates et al., 2015), and Satterthwaite approximations were applied to calculate the degrees of freedom (Satterthwaite, 1946; Kenny et al., 2002; Kuznetsova et al., 2017).

Similar to single-level mediation analyses, we conducted the multi-level mediation using a serial procedure similar to that applied by MacKinnon et al. (1995). This procedure involved: (1) determining whether the predictor variable influenced the outcome variable (path c); (2) determining whether the predictor variable significantly predicted changes in the mediator variable (path a); and (3) determining whether the mediator variable influenced the outcome variable (path b) when both predictor and mediator variables were included as predictors (path c'). As recommended for small samples, we used non-parametric bootstrapping procedures (see Preacher and Hayes, 2004; Preacher et al., 2007) to calculate the confidence intervals of path estimates in the mediation model. Mediation is significant in these analyses if the 95% bias-corrected and accelerated CIs for the indirect effect do not include 0 (Preacher and Hayes, 2004; Preacher et al., 2007).

## Results

### Pre-post Intervention Means and Standard Deviations

Table 2 presents the means and standard deviations at pre- and post-intervention for the student and teacher measures, along with the findings of pairwise *t*-test analyses. As shown, overall significant improvements were found from pre- to post-intervention on teacher measures of Distress, Personal Burnout, and Cognitive Flexibility (as measured by the Attention Switching Task overall Accuracy and Reaction Time). On the student measures, overall significant improvement was found on the SDQ Total Difficulties score. Perceived Teacher Support and Task Orientation also improved for students. Overall pre-post mean differences were not required for the pre-post difference to be considered for further analyses. The main reason for this is that the following analyses are interested in individual differences in the direction and magnitude of change rather than overall cohort differences across time.

**Table 2:** Time 1 (pre-intervention) and Time 2 (post-intervention) means and SDs for student and teacher measures, with corresponding pairwise *t*-statistics and *p*-values.

**Student**		**Time 1 Mean (SD)**	**Time 2 Mean (SD)**	**t(224)**	***p***
	SDQ total	10.20 (5.02)	9.58 (5.26)	2.45	0.015*
	ASP	23.80 (3.06)	23.80 (3.37)	−0.10	0.920
	WEMWBS	52.80 (7.36)	53.10 (8.14)	−0.65	0.514
	CES Innovation	6.68 (2.49)	6.56 (2.49)	0.82	0.411
	CES Affiliation	5.57 (1.83)	5.57 (2.08)	0.00	>0.999
	CES Teacher support	6.00 (2.19)	6.25 (2.32)	−1.82	0.070
	CES Task orientation	5.14 (1.78)	5.37 (2.00)	−1.86	0.064
	CES Order and organization	7.32 (2.58)	7.44 (2.65)	−0.87	0.384
	CES Rule clarity	5.23 (1.77)	5.03 (1.62)	1.64	0.101
**Teacher**		**Time 1** **Mean (SD)**	**Time 2** **Mean (SD)**	**t(16)**	***p***
	Distress DASS total	26.50 (18.00)	12.40 (7.29)	3.72	0.002**
	CBI Personal burnout	50.50 (22.00)	41.20 (16.90)	2.32	0.034*
	CBI Work burnout	52.90 (20.70)	45.00 (22.00)	1.95	0.069
	CBI Student burnout	33.10 (17.50)	27.50 (21.90)	1.37	0.189
	AST Accuracy	94.93 (4.91)	96.66 (5.57)	−2.26	0.038*
	AST RT	698.93 (83.82)	668.00 (80.84)	2.18	0.045*
	AST Switch RT	840.93 (122.43)	783.41 (109.59)	2.32	0.034*
	AST Non-switch RT	559.33 (81.71)	553.17 (83.74)	0.38	0.708

### Correlations: Student and Teacher Self-Report Measures

A post-intervention reduction in Distress (DASS total) was observed among teachers. Larger longitudinal reductions in teacher Distress (DASS Total) correlated with larger increases in the students' perceptions of Teacher Support as measured by the CES. Moreover, teachers evidenced a reduction in Personal, Work and Student-related Burnout following the intervention. Greater reductions in Personal Burnout (CBI Personal) correlated with greater increases of Academic Self-Perception (ASP) in students. Larger reductions in Work-related Burnout (CBI Work) in teachers also correlated with larger increases of Academic Self-Perception in students but was also related to larger decreases in perceived Teacher Support (CES Teacher Support). Finally, a greater reduction of Student-related Burnout in teachers (CBI Student) correlated with a larger reduction in the students' experience of Teacher Support (CES-Teacher Support). All correlational results can be found in Table 3.

**Table 3:** Correlations between pre-post-differences for teacher self-report and student self-report Measures.

		**Teacher Self-Report Measures**			
		Distress	Personal Burnout	Work Burnout	Student-Related Burnout
Student self-report measures	Total difficulties	0.112	−0.069	−0.033	−0.039
	Well-being	0.041	0.034	0.013	−0.019
	Academic self-perception	0.024	−0.17^*^	−0.148^*^	−0.111
	CES Innovation	0.023	−0.032	−0.127	−0.027
	CES Affiliation	0.045	0.007	−0.067	−0.079
	CES Teacher support	−0.21^**^	0.086	0.131^*^	0.136^*^
	CES Task orientation	0.009	0.037	−0.009	0.033
	CES Order and organization	0.028	−0.009	−0.074	−0.02
	CES Rule clarity	−0.066	0.026	0.056	0.028

* *p < 0.05*,

** *p < 0.01*,

*** *p < 0.001*.

### Correlations: Self -Report Measures and Teacher Cognitive Control Measures

Overall, a post-intervention reduction in response time (RT) and an increase in accuracy (ACC) was observed on the attention switching task (AST) of the CANTAB indicating an improvement in teachers' Cognitive Flexibility. Larger accuracy increases correlated significantly with a larger reduction of Distress (DASS Total) and with reduced Personal Burnout (CBI Personal). However, increased accuracy on the AST was also related to increased Student Burnout (CBI Client). Larger post-intervention decreases in reaction time speed (speed improvements) were related to greater reductions in Distress (DASS Total), yet smaller change in Work and Student burnout (CBI Work, Student). A breakdown of response time by switching versus non-switching trials is reported in Table 4. Increased student-reported affiliation was correlated with improvements in overall RT (and specific condition RT for non-switching trials). Increased perceptions of Teacher Support were related to overall improvements in RT (specifically too for switching trials), and also to improvements in accuracy. Despite being correlated with change in in Distress and Burnout, student Academic Self-Perception was not correlated with teacher Cognitive Flexibility. Teacher Support was the only student measure to be fully intercorrelated with teacher self-report measures and Cognitive Flexibility outcomes and was therefore included as the outcome measure in the mediation model below. All correlational results can be found in Table 4.

**Table 4:** Correlations between student and teacher self-report and teacher cognitive measures.

		**Attention Switching Task Outcomes**			
		**Overall Accuracy**	**Overall Reaction Time**	**Switching**	**Non-Switching**
Teacherself-reportmeasures	Distress (DASS Total)	−0.397***	0.145*	0.111	0.086
	CBI Personal burnout	−0.176**	0.002	0.091	−0.127
	CBI Work work burnout	−0.058	−0.117	−0.152*	0.012
	CBI Student burnout	0.296***	−0.158*	−0.214**	0.01
Studentself-reportmeasures	SDQ Total difficulties	−0.019	0.026	0.01	0.033
	Academic self-perception	−0.116	0.026	0.039	−0.01
	Well-being	0.036	0.021	0.052	−0.036
	CES Innovation	0.107	0.053	0.038	0.03
	CES Affiliation	0.023	−0.193**	−0.117	−0.158*
	CES Teacher support	0.17*	−0.163*	−0.16*	−0.054
	CES Task orientation	0.037	−0.085	−0.091	−0.014
	CES Order and organization	0.108	−0.007	−0.057	0.051
	CES Rule clarity	−0.016	−0.109	−0.164*	0.043

### Multi-Level Mediation Models

**Figure 1:**
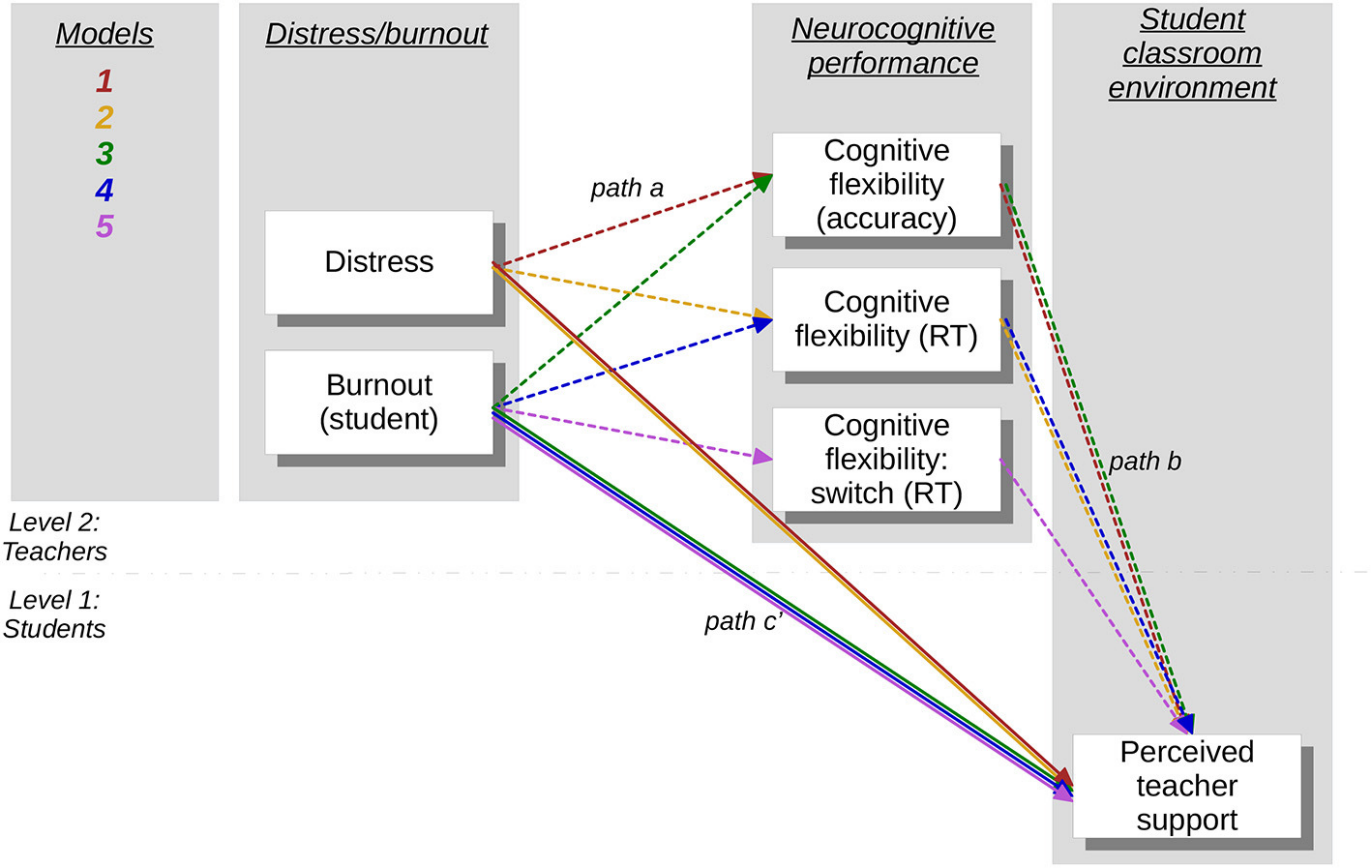
Illustration of the 2-2-1 mediation models proposed, with each model uniquely colored. All variables included reflect the change from pre- to post- intervention measurements.

The results of the correlation analyses revealed that Distress (DASS Total), and CES-Teacher Support factors were fully intercorrelated with Attention Switching overall accuracy (mediation 1), and mean reaction time (mediation 2). Additionally, Student-related Burnout and CES-Teacher Support were fully intercorrelated with overall accuracy on the attention switching task (mediation 3), mean reaction time (mediation 4), and reaction times for switch trials (mediation 5). The models are represented in Figure 1, and the results of each of these mediation models are reported below (see Table 5).

**Table 5:** Unstandardised path estimates (ß) with Standard Errors and bootstrapped 95% lower- and upper-confidence intervals in brackets.

**Model**	**Predictor**	**Mediator**	**Predictor-Mediator Path a**	**Mediator-Outcome Path b**	**Total Effect Path c**	**Direct Effect Path c'**	Indirect effect Path a*b
1	Distress (DASS Total)	AST Accuracy	−0.190, 0.029 [−0.248, −0.133]	0.064, 0.047 [−0.027, 0.156]	−0.059, 0.020 [−0.099, −0.019]	−0.050, 0.023 [−0.094, −0.006]	−0.012, 0.009 [−0.031, 0.005]
2		AST RT	1.201, 0.524 [0.168, 2.230]	−0.004, 0.003 [−0.009, 0.001]	−0.059, 0.020 [−0.099, −0.019]	−0.055, 0.020 [0.094, −0.016]	−0.005, 0.005 [−0.015, 0.002]
3	CBI Student-related burnout	AST Accuracy	0.070, 0.015 [0.040, 0.100]	0.081, 0.051 [−0.020, 0.181]	0.018, 0.011 [−0.003, 0.040]	0.014, 0.011 [−0.008, 0.035]	0.006. 0.004 [−0.001, 0.014]
4		AST RT	−0.611, 0.260 [−1.120, −0.097]	−0.003, 0.003 [−0.009, 0.002]	0.018, 0.011 [−0.003, 0.040]	0.015, 0.011 [−0.006, 0.037]	0.002, 0.002 [−0.002, 0.007]
5		AST Switch	−1.410, 0.422 [−2.240, −0.575]	−0.003, 0.002 [−0.007, 0.000]	0.018, 0.011 [−0.003, 0.040]	0.016, 0.010 [−0.004, 0.036]	0.004, 0.003 [−0.001, 0.011]

*N.B. All models tested included CES Teacher Support as the outcome variable*.

The results of mediation model 1 revealed that a reduction in Distress (DASS Total) scores was associated with increased accuracy on the AST task, and increased perceptions of Teacher Support; and the total effect was significant. However, the indirect effect was not significant suggesting that AST-Accuracy did not mediate the relationship between Distress and CES-Teacher Support. The direct effect revealed that, after accounting for accuracy in AST, a reduction in Distress (DASS Total) was directly predictive of increased CES-Teacher Support.

Mediation model 2 revealed that a decrease in Distress (DASS Total) was associated with a decrease in AST RT (improvement in performance). The total effect between Distress (DASS Total) and CES-Teacher Support was also significant, such that a larger reduction in Distress led to a greater increase in students' perceived Teacher Support (CES-Teacher Support). However, both the direct and indirect effects of Distress on CES-Teacher Support were not significant – suggesting that reaction time on the AST task did not mediate the relationship between Distress and perceived Teacher Support, and that when accounting for the influence of AST-RT, the direct effect of Distress did not significantly account for change in CES-Teacher Support.

The results of mediation models 3 through to 5 revealed no total effect of CBI client/student on CES-Teacher Support rendering the indirect and direct pathways non-significant (see Table 5).

## Discussion

In the present study, we explored the relationship between change in teacher perceived distress and burnout and students' well-being, academic self-perceptions, and evaluation of classroom environment. We aimed to assess whether change in teacher distress and burnout, following an 8-week stress-reduction intervention, resulted in downstream effects self-reported by students. A secondary aim of this study was to assess whether changes in teachers' cognitive flexibility mediated the relationship between teacher and student self-reported outcomes. As predicted, teacher stress and burnout affected multiple facets of students' well-being and the academic environment.

With regards to pre- and post-intervention scores for teacher and student measures, students reported a significant reduction in overall difficulties, and improved perceptions of teacher-support and task-orientation in the classroom environment. No change was indicated for students on measures of well-being, academic self-perceptions or other indicators of the classroom environment at this level of analysis. Teachers reported benefits of the intervention, with reductions in overall distress, and personal and student-related burnout. Additionally, following intervention, teachers saw marked improvements in their overall accuracy and reaction time on a cognitively demanding attention-switching task, with specific improvement recorded for switch trials of the task. This suggests that while there was an overall boost to speed and accuracy on the task, it seems that the intervention led to a specific improvement in effectively switching attention. Such improvements to switch-costs specifically have been reported for other well-being interventions (Wu et al., 2018), suggesting that targeted programs can not only enhance overall cognitive ability, but specifically enhance the core executive function of task-switching. A reduction in switch cost, also referred to as cognitive flexibility, can manifest as downstream performance improvement in problem-solving, decision making (Cañas et al., 2003; Hare et al., 2009) and multi-tasking; all of which are central to teaching. Greater cognitive flexibility is also known to drive the ability to change ones' own maladaptive beliefs and attitudes into more appropriate ones (Dennis and Vander Wal, 2010); again, a skill required for teaching.

In exploring the correlations between the teacher and student measures, improvements in teachers' cognitive flexibility as measured by task accuracy were associated with decreases in distress and personal burnout, and reaction time improvements were related to significant reductions in distress. Reduced symptoms of distress in teachers had a positive effect on student perceptions of teacher support. Furthermore, larger reductions in work and personal burnout in teachers was associated with greater improvements in students' academic self-perceptions. Critically, the results of this study indicate that changes in teachers' self-reported distress and burnout were consistently related to the students' perceptions of their teachers' support in the classroom. These findings are important because previous research indicates that students' perception of the teacher-student relationship predicts feelings of school belonging, perceptions of academic competence, and achievement in mathematics (Hughes, 2011). Further, the quality of the student-teacher relationship is an important predictor of students' school adjustment and behavioral/conduct problems (Baker et al., 2008). The research presented in this study suggests that decreasing teacher stress and burnout can improve the student-teacher relationship.

Interestingly, the present study indicated that cognitive flexibility in teachers did not mediate this aspect of the teacher-student relationships. Despite strong inter-correlations, performance on the attention switching task was not a mediating factor between teacher distress and student self-perceptions of teacher support. However, there was evidence of direct effects in the mediation model which suggests, after controlling for cognitive control/flexibility, the teachers distress score was predictive of students' perceptions of teacher support. This indicates that self-reported affective changes and cognitive flexibility in teachers independently contribute to the positive changes observed in students' academic self-perceptions. These results do not support the notion that the mechanism by which teacher stress and burnout affect students, is via neurocognitive performance. In other words, despite a reduction in distress and burnout predicting an improvement in accuracy and response time for cognitively demanding tasks, benefits perceived by students do not arise because of the improved cognitive flexibility of the teacher. Instead, our findings support a direct relationship between teacher stress and students' perceptions of the classroom. The research of Willis et al. (2019) highlights that teachers experience tension around balancing the academic agenda of the classroom with student well-being initiatives and managing mental health concerns in students. Although positive student well-being improves academic performance (e.g., Durlak et al., 2011), it is possible that teachers feel more capable of handling these concerns when their own emotional well-being is protected and supported.

Overall, the results of this study indicate that improving teachers' well-being has a downstream effect on students' perceptions of their teacher's supportiveness and a moderate impact on their academic self-perceptions and total difficulties in school. The results did not reveal any effect of neurocognitive mediation with regards to cognitive flexibility, however, other areas of cognition (i.e., attention and memory) may be potential factors of interest. Understanding how teachers' self-reported mental well-being acts to reduce or increase performance in students is critical to how teacher-student relationships are enacted.

Looking after teacher well-being is an extremely important and worthwhile endeavor. This has been particularly accentuated given the COVID-19 pandemic and the added workload pressures on teachers to provide continuity of learning to students through new online technologies, to support students remotely with their engagement, attendance, and behavior, and to be on the frontline for vulnerable children and young people (Gouedard and Pont, 2020). These exacerbating experiences may only add to the teacher attrition currently being experienced globally. As the present study has clearly demonstrated, teachers can be supported to find ways to relax, manage anxiety and build emotional resilience by training in mindfulness and compassion, and through exercise, music, and nutrition. They should be provided time to focus on their own mental health and well-being. Our current findings would suggest that educating teachers with strategies for managing stress will have positive top-down effects targeting the well-being of not only the teachers but also their students. However, such measures are only part of the solution to a complex problem. Teachers identify the sources of stress as usually systemic, pertaining to the job conditions themselves. Dealing with teacher stress must be a multi-factored approach and the wider systemic issues that create many of the sources of stress are critically important to be addressed through educational policy and school leadership (Chirico, 2017b).

The results of the present study provide important preliminary evidence that students benefit when their teachers' levels of stress and well-being meaningfully improve. Benefits are evident even after a relatively short period of time. Future studies are needed to further explore the flow-on benefits for students when their teacher's complete well-being programs using a rigorous study design including a control group, follow-up testing, mixed methods measures, and identification of factors that mediate and moderate outcomes for teachers and their students. Quality programs that address the well-being of teachers represent an exciting potential for positive downstream effects for students and beyond, underscoring the wide-reaching benefits of supporting our teachers in their essential and crucial daily task of educating and empowering the next generation of leaders.

## Data Availability Statement

The data that support the findings of this study are available from the corresponding author upon reasonable request.

## Ethics Statement

The studies involving human participants were reviewed and approved by the Human Research Ethics Committee of The University of Queensland. Written informed consent to participate in this study was provided by the participants' legal guardian/next of kin.

## Author Contributions

AC is Professor in the School of Education at The University of Queensland. She was the senior supervisor of this work. Her contributions included the conceptualization and design of the project, contributor to writing and editing, and providing critical feedback on the manuscript. AY was involved in conception and design of this work, analysis of data, and interpretation of research findings. She was also involved in data collection and interpretation of research findings and was an equal contributor to the writing of this manuscript. SF-C was involved in the conception and design of this work, analysis of data, interpretation of research findings and also equal contributor to writing this manuscript. ESC was critically involved in the conceptualization of the project, recruitment of participants, and all behavioral testing. She also was a contributor to the writing of this manuscript. LF is a post-doctoral research fellow and registered music therapist, her expertise in teacher-stress, well-being and early childhood was critical to the conception and design of this work. She was a contributor to the writing and provided critical feedback on the manuscript. JB is an educational consultant in emotional health and Honorary Research Fellow at UQ. She was involved in all aspects of project design and was able to contribute her knowledge and expertise during critical discussions on analysis and design. KF made substantial contribution to the conception and design of this work as well as recruitment of participants, and behavioral testing. MZ was involved in the collection of data and contributed her knowledge and expertise during critical discussions on analysis and design. All authors contributed to the article and approved the submitted version.

## Conflict of Interest

The authors declare that the research was conducted in the absence of any commercial or financial relationships that could be construed as a potential conflict of interest.
